# Supplementary figures and images for: Spectrum of clinical features and genetic variants in mevalonate kinase (MVK) gene of South Indian families suffering from Hyperimmunoglobulin D Syndrome
Source: PLoS One. 2020 Aug 21;15(8):e0237999. doi: 10.1371/journal.pone.0237999 (PMC7442240; doi:10.1371/journal.pone.0237999)

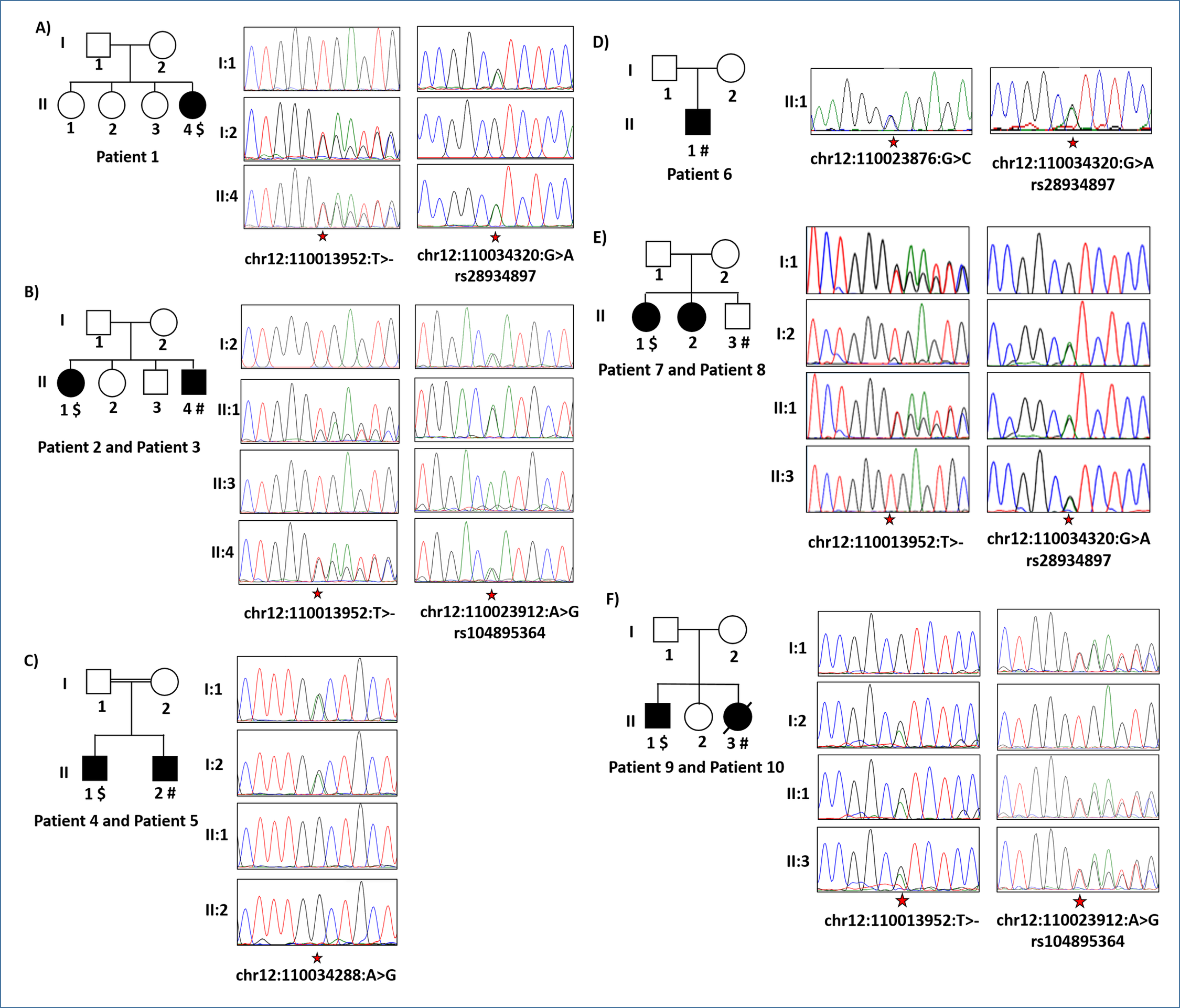

Supplement: S1 Fig — Samples marked $-were analysed using whole exome sequencing and marked # were analysed using Sanger capillary sequencing. (TIF) [file pone.0237999.s001.tif]
