# Supplementary material for: Spectrum of clinical features and genetic variants in mevalonate kinase (MVK) gene of South Indian families suffering from Hyperimmunoglobulin D Syndrome
Source: PLoS One. 2020 Aug 21;15(8):e0237999. doi: 10.1371/journal.pone.0237999 (PMC7442240; doi:10.1371/journal.pone.0237999)
Supplement: S1 File — (DOCX) [file pone.0237999.s004.docx]

**S1 File: Primer sequences for PCR amplification and Sanger sequencing with coordinates (GRCh37 or hg19)**

| **S. No.** | **Primer** | **Sequence** | **Coordinates (GRCh37 / hg19)** |
| --- | --- | --- | --- |
| 1 | Forward primer | GCCTCTGTGCTTATGTTTGC | chr12 + 110013707 110013726 |
|  | Reverse primer | TCTGTAGGCTCTTAGCACAC | chr12 - 110013975 110013994 |
| 2 | Forward primer | AGAGGTTCAGAGTGGACTTG | chr12 + 110023685 110023704 |
|  | Reverse primer | ACTCTTGGGCACCTACCATT | chr12 - 110023985 110024004 |
| 3 | Forward primer | TATGATGAGCTTCTCCCACG | chr12 + 110034168 110034187 |
|  | Reverse primer | CTGAAAAGGGAGAGAGAAGG | chr12 - 110034764 110034783 |

The conditions followed were denaturation at 95^o^C for 3 minutes, followed by 35 amplification cycles for 30 seconds. DNA denaturation is done at 95^o^C for 30 seconds, annealing at 56^o^C for 30 seconds, extension at 72^o^ C for 30 seconds and a final extension step at 72^o^C for 7 minutes.
