# Supplementary material for: Spectrum of clinical features and genetic variants in mevalonate kinase (MVK) gene of South Indian families suffering from Hyperimmunoglobulin D Syndrome
Source: PLoS One. 2020 Aug 21;15(8):e0237999. doi: 10.1371/journal.pone.0237999 (PMC7442240; doi:10.1371/journal.pone.0237999)
Supplement: Attachment — Submitted filename: Response to Reviewers.docx [file pone.0237999.s005.docx]

Journal Requirements:

When submitting your revision, we need you to address these additional requirements.

1. Please ensure that your manuscript meets PLOS ONE's style requirements, including those for file naming. The PLOS ONE style templates can be found at<https://journals.plos.org/plosone/s/file?id=wjVg/PLOSOne_formatting_sample_main_body.pdf> and<https://journals.plos.org/plosone/s/file?id=ba62/PLOSOne_formatting_sample_title_authors_affiliations.pdf>

**Response: Thanks for pointing that out, we have made the changes according to the PLOS ONE’s style.**

2. We note that you have indicated that data from this study are available upon request. PLOS only allows data to be available upon request if there are legal or ethical restrictions on sharing data publicly. For more information on unacceptable data access restrictions, please see<http://journals.plos.org/plosone/s/data-availability#loc-unacceptable-data-access-restrictions>.

In your revised cover letter, please address the following prompts:

a) If there are ethical or legal restrictions on sharing a de-identified data set, please explain them in detail (e.g., data contain potentially sensitive information, data are owned by a third-party organization, etc.) and who has imposed them (e.g., an ethics committee). Please also provide contact information for a data access committee, ethics committee, or other institutional body to which data requests may be sent.

b) If there are no restrictions, please upload the minimal anonymized data set necessary to replicate your study findings as either Supporting Information files or to a stable, public repository and provide us with the relevant URLs, DOIs, or accession numbers. For a list of acceptable repositories, please see<http://journals.plos.org/plosone/s/data-availability#loc-recommended-repositories>.

We will update your Data Availability statement on your behalf to reflect the information you provide.

**Response: We apologize for not making it clear. We have updated the cover letter with the information of data availability on request by the IGIB Institutional Ethics committee with their contact details.**

Comments to the Author

1. Is the manuscript technically sound, and does the data support the conclusions?

The manuscript must describe a technically sound piece of scientific research with data that supports the conclusions. Experiments must have been conducted rigorously, with appropriate controls, replication, and sample sizes. The conclusions must be drawn appropriately based on the data presented.

Reviewer #1: Yes

Reviewer #2: Yes

2. Has the statistical analysis been performed appropriately and rigorously?

Reviewer #1: N/A

Reviewer #2: N/A

3. Have the authors made all data underlying the findings in their manuscript fully available?

The [PLOS Data policy](http://www.plosone.org/static/policies.action#sharing) requires authors to make all data underlying the findings described in their manuscript fully available without restriction, with rare exception (please refer to the Data Availability Statement in the manuscript PDF file). The data should be provided as part of the manuscript or its supporting information, or deposited to a public repository. For example, in addition to summary statistics, the data points behind means, medians and variance measures should be available. If there are restrictions on publicly sharing data—e.g. participant privacy or use of data from a third party—those must be specified.

Reviewer #1: Yes

Reviewer #2: Yes

4. Is the manuscript presented in an intelligible fashion and written in standard English?

PLOS ONE does not copy or edit accepted manuscripts, so the language in submitted articles must be clear, correct, and unambiguous. Any typographical or grammatical errors should be corrected at revision, so please note any specific errors here.

Reviewer #1: Yes

Reviewer #2: Yes

5. Review Comments to the Author

Please use the space provided to explain your answers to the questions above. You may also include additional comments for the author, including concerns about dual publication, research ethics, or publication ethics. (Please upload your review as an attachment if it exceeds 20,000 characters)

Reviewer #1: This is a valuable contribution to the literature on HIDS (Hyper IgD Syndrome) or MVK deficiency. By presenting a small but by HIDS standard large case series with both clinical and molecular data it is a very useful contribution. The ten patients from six families represent the clinical spectrum previously reported with the additional information on the difficulty making the correct diagnosis and the other disorders that had been entertained before the molecular studies clearly identified the proper defect. Virtually nothing was said about outcome or treatment perhaps because the standard therapies that block IL1 (anakinra and canakinumab) are apparently unavailable or they plan to write another paper with that data. Surprisingly the authors state that the patients did well whereas untreated HIDS is typically associated with poor growth and development. None of the patients had the more severe form of the disease called mevalonic aciduria which is associated with severe and life threatening manifestations in infancy but not the periodic fever syndrome that HIDS displays.

**Response: We thank the reviewer for the suggestion. We have included more information regarding the clinical features, treatment, infection, previous diagnosis, development delay, deaths etc. for all the patients in the section on results and discussed them. The clinical features with other clinical details are summarized in the Supplementary Table 1.**

I liked tables 1 and 2 but not Figure 1 which I would like to replace with a linear model of the gene with the mutation sites indicated. Another figure which would be useful is a three dimensional model of the enzyme with the mutation sites identified. A little more focussed discussion of the deletion mutation and its effect on enzyme function or enzyme stability would have been interesting. The sequencing data is consistent with autosomal recessive inheritance requiring either homozygosity or compound heterozygote suggesting single mutations even if the deletion variant have enough residual activity to not be clinically symptomatic.

**Response: We thank the reviewer for their suggestion. We have included both the figures i.e. linear model of the gene with the mutation sites indicated. Also 3D model of the enzyme with mutation sites identified using PyMOL in Fig 1. We put the Sanger sequencing with pedigree figure in the supplementary figures. We have also discussed the deletion mutation effect on protein activity and stability in the Discussion part.**

Reviewer #2: Thank you for the opportunity to review this manuscript. Authors have described the clinical and molecular spectrum of Hyperimmunoglobulin D Syndrome. I have few comments-

Introduction-

Second para- 4th line- Authors could add more Indian references on MVK disorder. Some of the important references are as follows-

1: Correa ARE, Gupta N, Bagri N, Vignesh P, Alam S, Yamaguchi S. Mevalonate

Kinase Deficiency as Cause of Periodic Fever in Two Siblings. Indian Pediatr.

2020 Feb 15;57(2):180-181. PubMed PMID: 32060250

2: Jain A, Misra DP, Sharma A, Wakhlu A, Agarwal V, Negi VS. Vasculitis and

vasculitis-like manifestations in monogenic autoinflammatory syndromes. Rheumatol

Int. 2018 Jan;38(1):13-24. doi: 10.1007/s00296-017-3839-6. Epub 2017 Oct 14.

Review. PubMed PMID: 29032440.

3: Abdulla MC, Hawkins PN, Alungal J, Rowczenio D. Hyperimmunoglobulin D syndrome

in an Indian family undiagnosed for 11 years. Int J Rheum Dis. 2017

Dec;20(12):2236-2237. doi: 10.1111/1756-185X.12811. Epub 2015 Dec 1. PubMed PMID:

26620804.

4: Sinha A, Waterham HR, Sreedhar KV, Jain V. Novel mutations causing

hyperimmunoglobulin D and periodic fever syndrome. Indian Pediatr. 2012

Jul;49(7):583-5. PubMed PMID: 22885443.

**Response: We thank the reviewer for the suggestion, we have added papers from India in the manuscript.**

Methods-

Clinical work up- Whether any biochemical assays like GCMS was performed? In how many clinical diagnoses of Hyper IgD was made in the absence of IgD levels.

**Response: Unfortunately, GCMS was not done in any of the patients and IgD estimation was not possible in 70% of the children, although IgA levels were elevated in 100% of children. Total IgD was estimated in 2 patients and IgD surface in 1 patient which has been put in the Supplementary Table 1.**

Variant prioritization- Generally a MAF of 1% is used. The reason behind using MAF of 5% may be specified.

**Response: We appreciate the reviewer for pointing this out. We totally agree that in various studies, MAF was taken as 1% for variant filtering. But in a recent study by ClinGen experts showed that higher frequency (MAF>0.05) variants in one or more control populations could be pathogenic or likely pathogenic (PMID:31942019 PMID:30311383). So not to miss any variant that could be a pathogenic we kept the MAF>0.05 for variant filtering.**

Table 1- Elevated IgD is seen in patient 1 only.was it performed in one patient? or was it normal in others. I understand that it is not widely available.

**Response: As mentioned by the reviewer, total IgD estimation is not widely available in our country. Hence it could only be done only for two patients and Surface IgD could be done only for one patient.**

Information on consanguinity should be added in the table.

**Response: We thank the reviewer for the suggestion, We have added it to Supplementary Table 1 where only 1 family is consanguineous with homozygous mutation.**

Highlight the significance of elevated Vit B12

**Response: Unfortunately, we could not find any literature regarding elevated B12 levels in HIDS patients. However, in ALPS which is a well known primary immune deficiency disorder with elevated B12 levels, the reason attributed is an increase in the binding protein haptocorrin (PMID:22306884). Further studies will be necessary to ascertain this finding.**

Discussion-

Did authors find any genotype phenotype correlation.

**Response: We thank the reviewer for the suggestion, we have added the genotype-phenotype correlations in the discussion.**

Authors have stated that p.V377I and c.226+2delT are the common mutations in Indian subcontinent. This should be stated after incorporating the complete Indian data.

**Response: We thank the reviewer for this suggestion. We agreed that there should be complete Indian data to state the variant as a common mutation in Indian subcontinent. We have changed it to South India since all our patents belong to South India. All the variants which we found from the literature also had South Indian Ancestry.**

Was there any analysis done to prove the founder effect?

**Response: We are really thankful to the reviewer for that. We have put the GSA data and performed the identity by descent analysis of our two patients which had same recurrent compound heterozygous mutation to prove mutation to be a founder mutation.**

I think this line at the end of discussion ("In a patient who manifest ......... for V377I and c.226+2delT in MVK)may not be generalisable as Indian population is very heterogenous and this study mainly included the south Indian population .

**Response: We thank the reviewer for the suggestion, we agreed that the mutation could not be generalized to Indian population. So, we have made the changes of the mutation from Indian population to South Indian population.**

General comments-

Minor typos in introduction and discussion

**Response:** We have revised the manuscript and corrected typos.

6. PLOS authors have the option to publish the peer review history of their article ([what does this mean?](https://journals.plos.org/plosone/s/editorial-and-peer-review-process#loc-peer-review-history)). If published, this will include your full peer review and any attached files.

If you choose “no”, your identity will remain anonymous but your review may still be made public.

Do you want your identity to be public for this peer review? For information about this choice, including consent withdrawal, please see our [Privacy Policy](https://www.plos.org/privacy-policy).

Reviewer #1: No

Reviewer #2: No

[NOTE: If reviewer comments were submitted as an attachment file, they will be attached to this email and accessible via the submission site. Please log into your account, locate the manuscript record, and check for the action link "View Attachments". If this link does not appear, there are no attachment files to be viewed.]

While revising your submission, please upload your figure files to the Preflight Analysis and Conversion Engine (PACE) digital diagnostic tool,<https://pacev2.apexcovantage.com/>. PACE helps ensure that figures meet PLOS requirements. To use PACE, you must first register as a user. Registration is free. Then, login and navigate to the UPLOAD tab, where you will find detailed instructions on how to use the tool. If you encounter any issues or have any questions when using PACE, please email us at figures@plos.org. Please note that Supporting Information files do not need this step.
